# Visualization of Cytolytic T Cell Differentiation and Granule Exocytosis with T Cells from Mice Expressing Active Fluorescent Granzyme B

**DOI:** 10.1371/journal.pone.0067239

**Published:** 2013-06-28

**Authors:** Pierre Mouchacca, Anne-Marie Schmitt-Verhulst, Claude Boyer

**Affiliations:** 1 Centre d'Immunologie de Marseille-Luminy (CIML), Aix-Marseille University, UM2, Marseille, France; 2 Institut National de la Santé et de la Recherche Médicale (INSERM), U1104, Marseille, France; 3 Centre National de la Recherche Scientifique (CNRS), UMR7280, Marseille, France; Karolinska Institutet, Sweden

## Abstract

To evaluate acquisition and activation of cytolytic functions during immune responses we generated knock in (KI) mice expressing Granzyme B (GZMB) as a fusion protein with red fluorescent tdTomato (GZMB-Tom). As for GZMB in wild type (WT) lymphocytes, GZMB-Tom was absent from naïve CD8 and CD4 T cells in GZMB-Tom-KI mice. It was rapidly induced in most CD8 T cells and in a subpopulation of CD4 T cells in response to stimulation with antibodies to CD3/CD28. A fraction of splenic NK cells expressed GZMB-Tom ex vivo with most becoming positive upon culture in IL-2. GZMB-Tom was present in CTL granules and active as a protease when these degranulated into cognate target cells, as shown with target cells expressing a specific FRET reporter construct. Using T cells from mice expressing GZMB-Tom but lacking perforin, we show that the transfer of fluorescent GZMB-Tom into target cells was dependent on perforin, favoring a role for perforin in delivery of GZMB at the target cells’ plasma membranes. Time-lapse video microscopy showed Ca++ signaling in CTL upon interaction with cognate targets, followed by relocalization of GZMB-Tom-containing granules to the synaptic contact zone. A perforin-dependent step was next visualized by the fluorescence signal from the non-permeant dye TO-PRO-3 at the synaptic cleft, minutes before the labeling of the target cell nucleus, characterizing a previously undescribed synaptic event in CTL cytolysis. Transferred OVA-specific GZMB-Tom-expressing CD8 T cells acquired GZMB-Tom expression in Listeria monocytogenes-OVA infected mice as soon as 48h after infection. These GZMB-Tom positive CD8 T cells localized in the splenic T-zone where they interacted with CD11c positive dendritic cells (DC), as shown by GZMB-Tom granule redistribution to the T/DC contact zone. GZMB-Tom-KI mice thus also provide tools to visualize acquisition and activation of cytolytic function in vivo.

## Introduction

Cytolytic effector cells are of prime importance for protection by the immune system against pathogen infected or transformed cells. CD8 T lymphocytes and NK cells are the main effectors of perforin-GZM-dependent cytolysis. Naive CD8 T cells differentiate in secondary lymphoid organs upon encounter with cognate antigen presenting cells (APC) and become cytolytic T lymphocytes (CTL) after transcription and translation of genes encoding components of the cytolytic machinery, including perforin and GZMB. Once differentiated into effector CTL, CD8 T cells migrate to the tissues where their cytolytic machinery is activated upon encounter with cognate target cells. In contrast, resting splenic NK cells contain abundant amounts of the perforin and GZMB transcripts and, although the corresponding proteins are undetectable in them, these cells rapidly convert to functional killers upon culture in IL-2 or IL-15 [1].

For both CTL and NK cells, cytolytic effector proteins including perforin and various GZM are localized in cytoplasmic exocytic granules [2]–[5]. Perforin is a pore forming protein [6], [7] required for allowing GZMs to access target cells’ cytoplasm and induce apoptosis [8], [9]. Inactivation of the *Prf1* gene in mice has no consequences for the development of T lymphocytes, but severely affects cytolytic function [10] and immune responses [11], [12]. Indeed, when infected with LCMV, *Prf1 KO* mice develop a form of hemophagocytic lymphohistiocytosis [13] similar to the pathology affecting humans presenting mutations impairing perforin expression [14]. GZMB is part of the large family of serine proteases [15]. It has chymase activity and induces apoptotic cell death by cleaving, in particular, the pro-apoptotic hBid protein and the prodomain of caspase 3 [16]. The gene encoding GZMB [17] is localized on chromosome 14 in mice, within a cluster of genes encoding other GZM (C, F, G, D, E) [18]. GZMB is one of the most studied GZM, and is reported to be expressed in various cells of the innate and adaptive immune system (for review, see [5]). Inactivation of the *Gzmb* gene in GZMB-KO mice by 5′ insertion of the PGK-Neo cassette also led to reduction in *Gzmc, Gzmf, Gzmg* and *Gzmd* expression in Lymphokine Activated Killer (LAK) cells. Lymphoid development was not affected in these mice, but the extent of cytolysis and DNA fragmentation induced by CTL in target cells was decreased [18], [19]. GZMA and K encoding genes are localized on chromosome 13 in mice, and their expression is differently regulated from that of the GZMB group of genes in both T cells [20] and NK cells [1]. GZMA and GZMB reach the dense core of cytotoxic granules from the trans-Golgi network, while the pathway used by perforin is as yet un-characterized (reviewed in [2]). The dense core of the granules contains chondroitin sulfate proteoglycan covalently-linked to lattice-forming serglycin, which is necessary for the stability of the GZM, in particular GZMB, and of the granules [21]. The low pH (around 5) of the granules is inhibitory for perforin polymerization, so it remains inactive until released upon CTL degranulation. Cytolytic granules also contain inhibitors of GZM activity. These may contribute to the protection of the CTL from self killing [22]. The cytolytic granules belong to the lysosome family and express Lamp-1, Lamp-2 and CD63 [23]. GZM have to undergo a maturation step that for GZMB involves cathepsin C and H [24]–[26].

Killing of pathogen-infected target cells results from the delivery of CTL cytolytic granule contents at the immune synapse generated upon the cognate recognition of the target by the CTL. Generation of a stable CTL-target cell interaction requires engagement of the clonally expressed CTL T cell receptor (TCR) with cognate peptide/MHC complexes on the target, as well as stabilization of that interaction by CTL-expressed LFA-1 engaging target-expressed ICAM molecules. This engagement results in TCR/CD3 signaling activating protein tyrosine kinases, initiating a series of activation pathways. Among these, phosphorylation of PLCγ induces the activation of PKCs as well as Ca++ influx that are involved, respectively, in mechanisms of granule polarization [2], [27]–[29] and exocytosis [30]. Upon CTL/target cell contact, changes in the CTL cellular shape appear and a stable contact zone is established between the two cells forming the immune synapse. Next, the cytolytic granules migrate along the microtubules, are polarized towards this contact zone [31]–[33] and dock at the CTL plasma membrane. Upon fusion with the plasma membrane, the granules release their contents. This step leads to Lamp-1 and 2 exposure at the CTL cell surface before their re-internalization into the CTL [34], [35]. Thus, granule polarization towards the CTL/target immune synapse signals the first step of the activation of the CTL cytolytic process. Subsequent steps involve release of the GZM and perforin from the CTL by exocytosis and their entry into the target by a mechanism that is still debated [36]–[38]. Finally, GZM’s protease activities induce target cell death by apoptosis.

To optimize CTL responses against pathogens it is important to be able to monitor the distinct steps of acquisition and activation of their cytolytic function during an immune response. With that aim and based on our previous experience [39], we have constructed by homologous recombination a KI mouse genetically modified to express a fusion protein between GZMB and tdTomato (GZMB-Tom), a powerful red fluorescent tandem dimer protein [40], in place of GZMB, maintaining the gene expression control elements of the endogenous *gmzb* gene. This mouse, called GZMB-Tom-KI, has normal lymphocyte development and distribution. We show that the acquisition of GZMB expression in subpopulations of lymphocytes from these mice can be faithfully monitored ex vivo or in vivo by measure of tdTom fluorescence by FACS or fluorescence microscopy.

We also show that GZMB-Tom localized to CTL granules and was an active protease when degranulated into cognate target cells, as shown using target cells expressing the FRET reporter construct CFP-DEVD-YFP [41]. By FACS analysis GZMB-Tom exocytosis from the CTL and delivery in the target cells could be monitored, respectively, by decreased and increased tdTom fluorescence in conditions of physiological expression levels of GZMB.

Using time-lapse video microscopy, it was possible to show sequential events of Ca++ signaling in CTL upon their interaction with cognate targets and relocalization of GZMB-Tom-containing granules to the synaptic contact zone. This was followed by a perforin-dependent entry of the non-permeant dye TO-PRO-3 at the synaptic cleft, an event that could be visualized minutes before TO-PRO-3 staining of the dying target cell nucleus.

Upon transfer into Listeria monocytogenes-OVA infected mice, OVA-specific GZMB-Tom -KI CD8 T cells (GZMB-Tom-KI-OT1 CD8 T cells) acquired GZMB-Tom expression as soon as 48h after infection. These GZMB-Tom positive CD8 T cells localized in the splenic T-zone where they interacted with CD11c positive DC, as shown by GZMB-Tom granule redistribution to the T/DC contact zone.

In conclusion, the GZMB-Tom-KI mice provide accurate tools to visualize acquisition and activation of cytolytic functions by various immune effector cells in vitro and in vivo.

## Materials and Methods

### *Granzyme B-tdtomato* Gene Constructs and Production of Knock In (KI) Mice

A 8051 bp region (Fig.S1A, regions A and B on the scheme) was isolated in a pBS-KS vector from a BAC clone (RP23-38D2) containing the entire *Gzmb* gene plus 196kb upstream and 21kb downstream from the start of the coding region [18]. Multiple enzymatic sites (Fse I, Not 1, Eco RV and Sal I) were included throughout the construct by using ET-recombination [42]. These sites allowed for insertion by digestion/ligation of the *tdTomato* cDNA [40] completed by a portion of the 5′ final exon of *Gzmb* and a 12 nucleotide sequence encoding a linker which was extracted from a previous construct. A self Deleter Neo cassette (auto deletion in F0 male gametes) and a counter selection TK Cre cassette were inserted using the same method (Fig.S1A). ES cells of C57BL/6 (B6) origin were transfected by electroporation and clones having recombined *Gzmb-tdTom* were selected by antibiotic resistance. Figure S1B shows the recombinant locus in modified ES cells (***Gzmb-tdTomato Neo***) and final locus (***Gzmb-tdTomato*** **Δ** ***Neo***) in *Gzmb-tdTomato Knock-In* (KI) mice. Final verifications of recombinant ES clones performed by Southern Blot and by PCR 5′/3′ screens showed a correct insertion of the modified *Gzmb-tdTomato* locus at the expected genomic region and the NEO screen confirmed its unique insertion (Fig.S1C).

### Gzmb-tdTomato Mouse Genotyping

**Figure 1:**
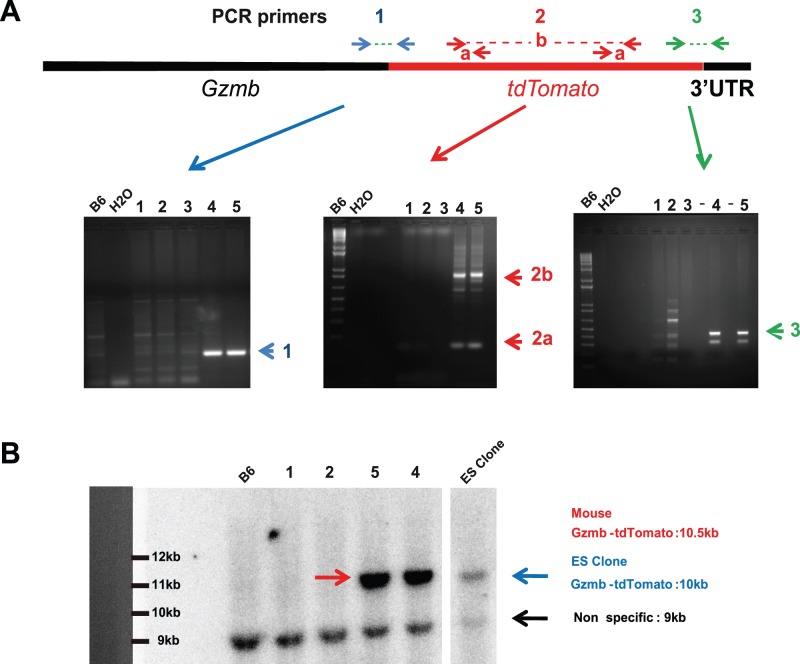
Construction and screening of GZMB-Tom-KI mice. Construction of the vector used for homologous recombination in the *Gzmb* locus and characterization of a recombinant ES clone of C57BL/6 (B6) origin with integration of the construct containing the *gzmb* gene, deleted of its stop codon, followed by a sequence encoding a 12 amino acid linker followed by the *tdTomato* cDNA sequence are described in Material and Methods and Figure S1. Offspring (1–5) from a chimeric mouse (obtained from Balb/c mice blastocysts injected with the ES *Gzmb-tdTom* recombined clone) crossed to B6 mice was screened by PCR with a B6 negative control (A) or by Southern blot with a B6 negative control and a recombined ES cell as a positive control (B). Raw mapping of the *gzmb-tdtomato* allele performed by multiple PCR (see Materials and Methods) revealed offspring 4 and 5 as positive for PCR1 (blue), PCR2 (red) and PCR3 (green) (A). A Southern Blot using a ^32^P labeled tdTomato cDNA probe revealed the presence of the *tdTomato* sequence in genomic DNA from offspring 4 and 5 (10.5kb) and in the recombined ES clone (10kb).

Balb/c mice blastocysts were injected with the ES *Gzmb-tdTom* recombined clone. Chimeric mice were crossed to B6 mice and the black offspring were tested. Genomic mapping of the *Gzmb-tdTomato* allele was analyzed by PCR using primers encompassing the Gzmb and tdTomato sequences (PCR1), primers within the tdTomato sequence (PCR2) and primers encompassing the tdTomato and 3′UTR Gzmb sequences (PCR3) (Fig. 1A). The product from PCR3 was sequenced by the Sanger method and showed 100% match with the expected sequence after auto-deletion of the NEO cassette. A Southern Blot using a ^32^P labeled probe generated from tdTomato cDNA was performed on DNA from B6 wild type and from the offspring of the cross between chimeric and B6 mice (Fig. 1B). Enzyme digestion with Hind III was such that a 10.0 kb and 10.5 kb was expected for ES cells (*Gzmb-tdTomato Neo*) and Gzmb*-tdTomato* mice, respectively (Fig. 1B). The heterozygous matings with *Gzmb-tdTomato* mice produced the expected numbers of heterozygous and homozygous mutant progeny (Mendelian frequencies); these mice developed normally and were fertile and were indistinguishable from their wild-type littermates.

Genotyping of the *Gzmb-tdTomato* locus used the following primers for PCR screening: *Gzmb* FW (forward primer), 5-CATGCTTCTCTGGGAGGAGCC-3; *tdTomato* FW, 5-CACCTGTTCCTGTACGGCATGG-3 and *Gzmb-(tdTomato)* 3′ UTR (RV primer), 5-ATTCTCGGGGCACTGAGGGG-3. The PCR protocol involves 94°C for 2 min, 45 cycles of 58°C for 30 s, and 72°C for 30 s completed by 72°C for 5 min. The PCR product is 297 bp for the *Gzmb-tdTomato* allele and of 510 bp for the wild-type allele.

### Mice and Ethics Statement

All procedures were approved by the Regional ‘‘Provence-Alpes-Cote d’Azur’’ Committee on Ethics for Animal Experimentation (authorization: n°13.521, date: 08/10/2012) and were in accordance with French and European directives. All efforts were made to minimize animal suffering.

Mice were housed under specific pathogen-free conditions in the CIML facility. *Gzmb-Tom-KI* mice (GZMB-Tom-KI) were backcrossed 3 times to B6 mice, before their interbreeding to obtain mice homozygous for the mutant allele (GZMB-Tom-KI/KI). Mice homozygous for the mutation appeared indistinguishable from their heterozygous littermates and both types of mice have been used in this study. GZMB-Tom-KI/KI mice were also crossed with OT1-TCR transgenic mice (TCR that recognizes the OVA-peptide SIINFEKL/H-2K^b^, hereafter referred to as OT1 mice), purchased from the Jackson Laboratory, generating GZMB-Tom-KI OT1 mice, and further GZMB-Tom-KI/KI-OT1 mice. GZMB-KO mice, a kind gift from M. Simon (Max-Planck-Institut für Immunbiologie, Freiburg, Germany), were also crossed with OT1 mice. Perforin deficient (Perf-KO) mice, were obtained from the Jackson laboratory (strain C57BL/6-*Prf1* ^t*m1Sdz*^/J) and were crossed with the GZMB-Tom-KI/KI to obtain Perf-KO-GZMB-Tom-KI/KI-OT1 mice.

### Cells

Tumor lines used as target cells included RMA-S (tap1−/− thymoma, H-2^b^, obtained from the American Tissue Culture Collection (ATCC)), OVA-expressing EL4 thymoma (EG7) [43] and EG7 cells expressing the FRET reporter construct CFP-DEVD-YFP (EG7-DEVD) allowing for detection of GZMB activity (directly or indirectly via its activation of caspase 3) by measure of FRET disruption [41], T cell purification from spleens or LN was performed by negative selection using Dynabeads (Dynal).

### Antibodies and FACS Analysis

Cells in suspension were labeled with classical mAb from BD, Pharmingen or e-Bioscience and were analyzed with a LSRII FACS equipped with a 561 nm laser. GZMB-Tom was analyzed at the G610 channel as described [39] and this fluorescence is designated as tdTom.

### Cell Culture

Lymphocytes from different organs were collected from GZMB-Tom-KI/KI, GZMB-Tom-KI or their WT littermates or C57BL/6 mice. Stimulation of T cells (0.5 10^6^cell/well in 24 well culture plates), by plastic-coated anti-CD3 mAb (145.2C11, 10 µg/ml PBS) was performed in the absence or presence of soluble anti-CD28 mAb (2 µg/ml added at the beginning of the culture). For generation of Cytolytic T lymphocytes (CTL), CD8 T cells cultured for 3 days on anti-CD3 and CD28 mAb, as above, were further expanded in IL-2 (10 U/ml) for about one week, becoming enriched for CTL. For some cultures, CD8 T cells were pre-loaded with 2.5 µM CellTrace™Violet (CTV, Molecular Probes) as described by the manufacturer. Splenocyte cultures for NK cell stimulation used 3.10^6^ cells in the presence of 1000U mouse IL-2, as described [1]. To obtain CTL from OT1-TCR-transgenic mice, CD8 T cells (0.5 10^6^/well of a 24 well culture plate) were added to 10^5^ syngeneic irradiated splenocytes loaded with 0.1 µM SIINFEKL peptide. After 3 days cells were expanded with 10 U/ml IL-2, and kept for about one week.

### Characterization of the GZMB-Tom Fusion Protein by Western Blot

Immunoblots to define the MW of the GZMB-Tom fusion protein expressed in GZMB-Tom-KI/KI CTL were performed as described [39]. A protein of around 80kDa was recognized by the anti-RFP Ab that cross-reacts with tdTom (Fig.S2). This MW corresponds to the 29 kDa for GZMB added to the 50 kDa of tdTomato.

### Analysis of Lamp-1 Exposure during CTL Degranulation

As previously described [39], CTL and target cells (RMA-S cells loaded with either the relevant OVA SIINKEFKL or irrelevant pKB1 peptide or EG7-DEVD cells) were mixed at different effector to target ratios (1/1 or 3/1) in the presence of anti-Lamp-1/CD107 mAb [34] for 1–2h at 37°C. Labeling with anti-CD8 mAb was then performed before FACS analysis at the LSRII-561.

### Measure of Degranulated GZMB-Tom Activity in vitro

WT, GZMB-Tom-KI/KI or GZMB-KO/KO, or Perf-KO/KO-GZMB-Tom-KI/KI CTL (10^6^) were activated during 4h with coated anti-CD3 mAb or Ionomycin (2 µg/ml) and PMA (50 ng/ml). Supernatants were tested for GZMB protease activity after capture with anti-GZMB Ab using the Human Granzyme B activity test from Quickzyme Bioscience.

### Evaluation of GZMB-Tom Activity in the EG7-DEVD Target Cell by FRET Disruption

Target cells are thymoma EL4 cells expressing the antigen OVA (EG7) as well as the FRET-based fluorescent probe CFP-DEVD-YFP (EG7-DEVD). Cleavage of the probe directly by GZMB or by caspase 3 activation leads to disruption of the FRET. For analysis, the LSRII FACS was used with the following settings: excitation of CFP with the laser at 405 nm, and collection of fluorescence emissions at the Pacific Blue channel (455/30 nm) for CFP, and Amcyan channel (525/50 nm) for YFP. CTL were from WT-OT1, GZMB-Tom-KI/KI-OT1 and GZMB-KO/KO-OT1 mice**.** CTL specific degranulation during the test was measured by anti-Lamp-1 mAb uptake and tdTomato fluorescence.

### Confocal Microscopy Analysis

Fixed or fixed and permeabilized cells were prepared for confocal analysis as described [39] and analyzed using a Leica SPX5 or Zeiss780 confocal microscope.

### Video Microscopy Analysis

CTL were labeled with Fluo-4 acetoxymethylester (AM) (Mol. Probes) allowing for monitoring of Ca++ signaling [39]. Target cells were pre-loaded with calcein violet AM (1 µM final) (Mol. Probes). The cell permeant AM dyes become fluorescent after cleavage of the acetoxymethyl group by intracellular esterases. Permeabilization of the target cells’ plasma membrane can be observed by calcein leakage, as described [44]. TO-PRO-3 (1/500–1/2000) (Mol. Probes), a non-permeant double stranded DNA intercalating dye, was present in the medium throughout the experiment, allowing for detection of its binding to DNA of apoptotic target cells. For video microscopy analysis, RMA-S target cells pre-incubated with 1 µM OVA peptide and loaded with 1 µM calcein AM were deposited onto slides (poly-lysine activated borosilicate glass bedded n°1 wells (Lab-Tek, Nunc) in RPMI medium. CTL were next deposited in RPMI-5% FCS medium without phenol red containing TO-PRO-3. Time Lapse analysis was performed using a Zeiss780 confocal microscope (40X oil objective and a 0.7 aperture, pinhole 8 µM).

### In vivo Immunizations and Analysis

CD8 T cells from GZMB-Tom-KI-OT1 or WT-OT1 mice, both CD45.2, were purified from LN, and loaded with 2.5 µM CTV before i.v. injection (3.10^6^ cells) into C57BL/6 recipient mice (CD45.1). Half of these mice received 10.000 U Listeria-OVA [45] by i.v. injection 11h later and spleens were recovered from immunized and non-immunized mice as indicated. FACS analysis was performed on fixed splenocytes (from 1/3 of each spleen). Immunohistology was performed on sections of the same spleen after fixation with 2% PAF solution in a Phosphate Buffer containing L-Lys and sodium periodate for 24h at 4°C, followed by a wash for 1h in phosphate buffer and 12h incubation in 30% sucrose at 4°C before embedding in OCT as described [45]. Spleen sections were analyzed using a Zeiss780 confocal microscope after labeling with Ab as described in the figures.

### Statistics

Statistical analyses were performed with the Student’s t test using GraphPad and two-tailed P values are given as: (*) P<0.05; (**) P<0.01and (***) P**<**0.001; (ns) P>0.05.

## Results

### T and NK Cell Composition in Lymphoid Organs of GZMB-Tom-KI Mice

**Figure 2:**
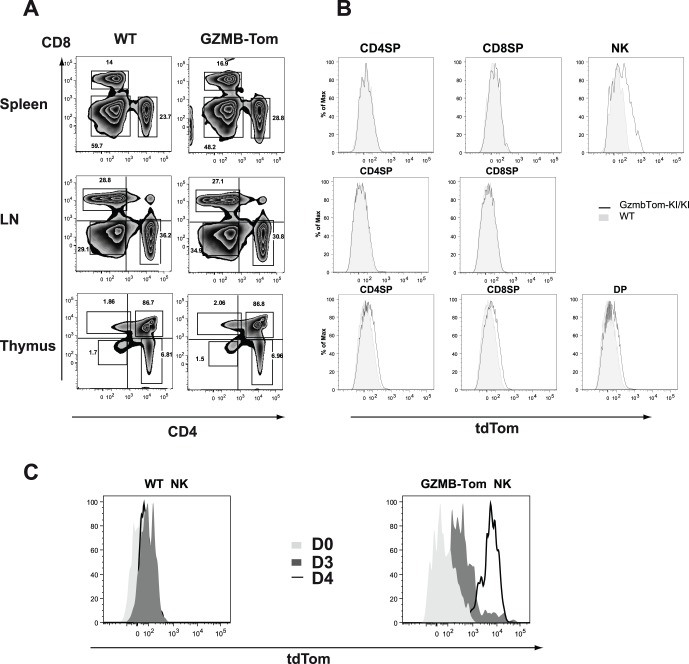
Comparison of T and NK cell composition in the lymphoid organs of C57BL/6 and GZMB-Tom-KI mice. FACS analysis of CD4 and CD8 T lymphocytes in spleen, lymph nodes (LN) and thymus (A, B) and of NK cells in spleen (B) from C57BL/6 (WT) and from GZMB-Tom-KI/KI mice. In B, level of GZMB-Tom expression is shown on CD4 and CD8 T lymphocytes and on NK cells (spleen) or CD4CD8 double positive (DP) cells (thymus) from WT (filled in grey) and GZMB-Tom-KI (black line) mice. In C, splenocytes were cultured with 1000U/ml IL-2 and analyzed before culture (D0) and 3 (D3) and 4 (D4) days later for GZMB-Tom expression on NK cells, defined as NK1.1+ CD3- cells. One representative experiment of at least two experiments is shown.

Comparing GZMB-Tom-KI/KI, GZMB-Tom-KI/WT and WT mice, we observed similar sizes of lymphoid organs (not shown). The distribution of CD4, CD8 T cells (Fig. 2A) and NK cells (not shown) in spleen, lymph nodes (LN) and thymus (Fig. 2A), as well as in blood (not shown) was also similar for WT and GZMB-Tom-KI/KI mice. Analysis of GZMB-Tom expression (see Materials and Methods) revealed that CD8 and CD4 T cells as well as thymocytes failed to express GZMB-Tom when directly taken from mice. Some splenic NK cells by contrast were slightly positive (Fig. 2B). NK cells represented 10–13% of splenocytes and among these 7–10% were GZMB-Tom positive with a tdTom mean fluorescence intensity (MFI) of 584 for NK cells from GZMB-Tom-KI/KI mice compared to MFI 31 for NK cells from WT mice (Fig. 2B). Resting splenic NK cells are poorly cytotoxic and contain little GZMB, but can be induced to express abundant GZMB in response to IL-2 in vitro [1]. In agreement with this report, we observed that upon culture of GZMB-Tom-KI/KI splenocytes with 1000 U/ml IL-2, 24% of NK cells expressed high tdTom fluorescence (MFI 5371) after 3 days, and 90% of them were highly positive (MFI 6666) after 4 days (Fig. 2C). By contrast tdTom fluorescence was not observed for IL-2 cultured WT NK cells (MFI tdTom 65).

### In vitro Acquisition of GZMB-Tom Expression by CD4 and CD8 T Cells in Response to CD3 and CD28 Engagement

**Figure 3:**
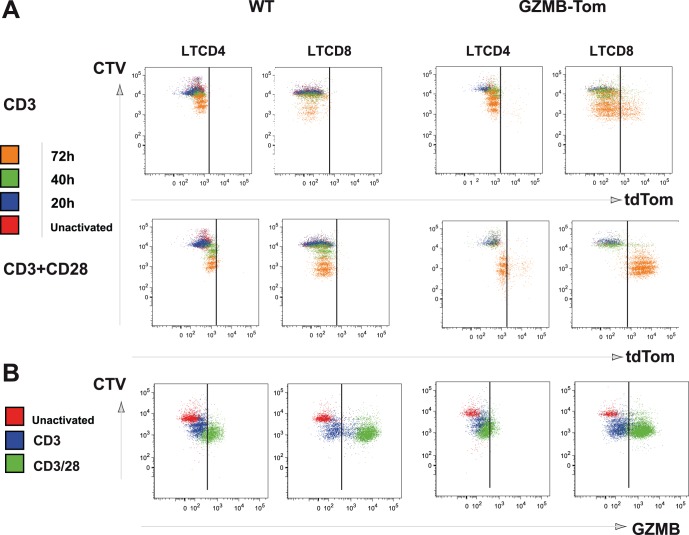
GZMB-Tom and GZMB expression as a function of T cell division in culture. T cells purified from LN of C57Bl/6 (WT) or GZMB-Tom-KI/KI mice pre-labeled with 2.5 µM CTV were cultured (0.3 10^6^/well) on costar plates (24-well) pre-coated with anti-CD3 10 µg/ml with additional soluble anti-CD28 (2 µg/ml final) (a-CD3/28) or not (a-CD3). **A**: GZMB-Tom expression (tdTom) is shown in CD4 and CD8-gated T cells before (Unactivated) and after 20h, 40h or 72h in culture as a function of cell division (CTV). **B**: In parallel, GZMB expression as detected with anti-GZMB mAb (GZMB) on fixed and permeabilized cells is shown before (Unactivated) and 72h after culture with anti-CD3 or anti-CD3/CD28 as in (A). One experiment representative of 3 independent experiments is shown.

To test whether acquisition of tdTom fluorescence faithfully reproduced the induction of GZMB in CD4 and CD8 T cells, T cells purified from LN of WT and GZMB-Tom-KI/KI mice were labeled with CTV to follow cell division, and stimulated with coated anti-CD3 mAb (145.2C.11) in the absence or in the presence of costimulation by soluble anti-CD28 mAb (57.1) (see Materials and Methods). Cells were analyzed daily for different cell surface receptors (TCR, CD44, CD69, CD25, CD4, CD8, not shown), and for GZMB-Tom expression as a function of cell division (Fig. 3A). A comparison with GZMB expression as detected by anti-GZMB Ab on fixed and permeabilized cells is also shown (Fig. 3B).

For all T cells, the first division was observed at 40h (Fig. 3A-green) and 4–5 divisions had occurred at 72h (Fig. 3A-orange). Activation with anti-CD3 alone (Fig. 3A line 1) led to division of approximately 80% of CD4 and of CD8 T cells at 72h, for both WT and GZMB-Tom-KI/KI T cells. Activation with anti-CD3/28 increased cell divisions as more cells (about 95%) reached the 5^th^ division at 72h (Fig. 3A, line 2 orange), for all T cells. When activated by anti-CD3 or anti-CD3/28, no WT CD4 or CD8 T cells exhibited tdTom fluorescence, as expected. The expression of GZMB-Tom in GZMB-Tom-KI/KI CD4 and CD8 T cells is represented in Figure 3A (columns 3 and 4). For CD4 T cells activated with a-CD3 alone, very few cells (2.8%) expressed GZMB-Tom at 72h (Fig. 3A, line 1, column 3). Activation with a-CD3/28 increased expression of GZMB-Tom in CD4 T cells at 72h (13%, Fig. 3A line 2, column 3), whereas it was undetectable at 40h. For GZMB-Tom-KI/KI CD8 T cells the expression of GZMB-Tom was higher than in CD4 T cells, as expected. When stimulated with a-CD3 alone, very few CD8 T cells expressed GZMB-Tom before the 1^st^ division (Fig. 3A, line 1 column 4, blue). As cells divided, more CD8 T cells acquired the expression of GZMB-Tom, reaching 24% at 72h (with MFI tdTom 4240). When stimulated with a-CD3/28, almost all GZMB-Tom-KI/KI CD8 T cells became positive for GZMB-Tom expression (94%) and tdTom MFI reached 7053.

We next analyzed the expression of GZMB at day 3 using anti-GZMB Ab. For GZMB-Tom-KI/KI T cells, detection of GZMB-Tom in both CD4 and CD8 T cell subsets was similar whether tdTom fluorescence was measured (Fig. 3A) or anti-GZMB Ab was used (Fig. 3B). In addition, the data showed that WT T cells expressed GZMB with kinetics and percent positive cells (Fig. 3B columns 1–2) similar to those observed for GZMB-Tom-KI/KI T cells.

Thus, upon stimulation with anti-CD3 alone, for GZMB-Tom-KI/KI cells, 3.7% of CD4 T cells and 20% (MFI 881) of CD8 T cells were GZMB positive, whereas for WT cells no GZMB positive CD4 T cells were observed and 28% (MFI 2386) of CD8 T cells were positive. When GZMB-Tom-KI/KI cells were stimulated with anti-CD3/CD28, 32% (MFI 493) of CD4 T cells and 95% (MFI 1745) of CD8 T cells were GZMB positive. For WT cells, 58% (MFI 810) of CD4 T cells and 95% (MFI 6736) of CD8 T cells were positive. Note that the GZMB level of GZMB-Tom-KI/KI T cells is lower (MFI 1745 compared to 2386 for CD8 T cells for instance). This may be attributable to a lower affinity of the anti-GZMB Ab to the GZMB-Tom fusion protein.

In conclusion, we observed that the GZMB-Tom-KI/KI T cells expressed GZMB-Tom in a manner similar to GZMB expression in WT T cells when cultured in vitro with anti-CD3 or anti-CD3/28 mAb. CD8 T cells expressed higher levels of GZMB or GZMB-Tom than CD4 T cells. The CD28 costimulatory signal led to increased GZMB or GZMB-Tom expression and cell division as expected [46] both for CD4 and CD8 T cells.

### Analysis of the Intra-cellular Distribution of GZMB-Tom by Confocal Microscopy

**Figure 4:**
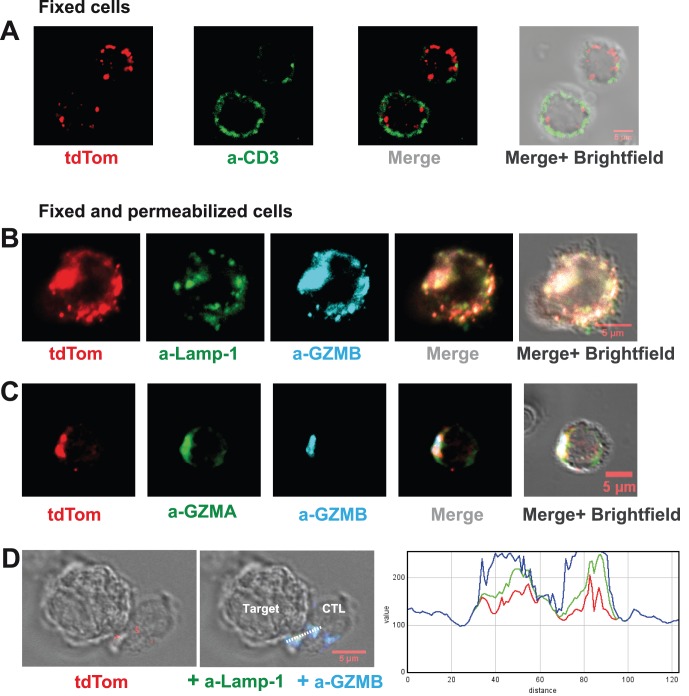
Analysis of GZMB-Tom-KI distribution in CTL by confocal microscopy. GZMB-Tom-KI CTL were produced from LN T cells by a-CD3/CD28 stimulation (as in Fig. 3) followed by IL-2 expansion for analysis at day 8 of culture and treated as described in Materials and Methods. A: Cells were labeled with anti-CD3 (17A2-A647) before fixation and single fluorescence of tdTom (red) or anti-CD3 (green), as well as merged fluorescence images are shown. B-D: Cells were fixed and permeabilized for labeling with antibodies diluted in Saponin buffer. Labeling with a-CD107a (Lamp-1, 1D4B-A488, green) and a-GZMB (MHGB05-APC, blue) (B, D) or a-GZMA (3GB8.5-FITC, green) and a-GZMB (blue) (C) is shown as well as tdTom fluorescence (red) (B-D). In (D) CTL were derived from GZMB-Tom-KI/KI-OT1 mice (see Methods) and incubated for 25 min at 37°C with RMA-S target cells loaded with the OVA peptide (10^−6^ M). Histograms of an RGB line profile show colocalization of tdTom (red), a-Lamp-1 (green) and a-GZMB (blue). A-D: For evaluation of colocalization, variations of two fluorescence intensities were compared and Rr Pearson's coefficients were calculated (as in [39], and see Fig.S3). Rr = 0 (no co-localization) for CD3/tdTom (A); Rr around 0.65 (good colocalization) for tdTom/Lamp-1, Lamp-1/GZMB and tdTom/GZMB (B); Rr = 0.45 for both tdTom/GZMA and GZMA/GZMB and 0.60 for tdTom/GZMB (C); Rr around 0.7 for all 3 couples tdTom/Lamp-1, Lamp-1/GZMB and tdTom/GZMB in the CTL/target conjugates (D).

The intra-cellular distribution of GZMB-Tom was analyzed by confocal microscopy on CTL following activation with anti-CD3/28 in culture and expansion in IL-2 (see Material and Methods). In fixed cells labeled with anti-CD3 mAb (green), GZMB-Tom (red) is localized in granules throughout the cytoplasm, whereas CD3 is localized at the external membrane (Fig. 4A). In Figure 4B-C, fixed and permeabilized cells were labeled with anti-Lamp-1 and anti-GZMB mAb (B) or anti-GZMA and anti-GZMB mAb (C). Colocalization of tdTom fluorescence was observed with GZMB and GZMA in Lamp-1-expressing vesicles (see Fig.S3 for Pearson’s coefficients). In conclusion, GZMB-Tom in CTL from GZMB-Tom-KI/KI mice is located in true cytolytic granules coexpressing GZMB-Tom with Lamp-1 and GZMA. As expected, the GZMB-Tom labeled granules move towards the CTL/target contact zone and colocalization of tdTom fluorescence with GZMB and Lamp-1 labeling is maintained (Fig. 4D and Fig.S3). Note that although optimal conditions were used to analyze GZMB-Tom by confocal microscopy [39], a loss of tdTom fluorescence was observed when cells were permeabilized. The use of fluorescent proteins like tdTom is thus particularly suitable for the observation of living cells, but fixation and especially permeabilization leads to an important reduction in fluorescence.

### GZMB-Tom Degranulation from CTL and Perforin-dependent Transfer in Cognate Target Cells

**Figure 5:**
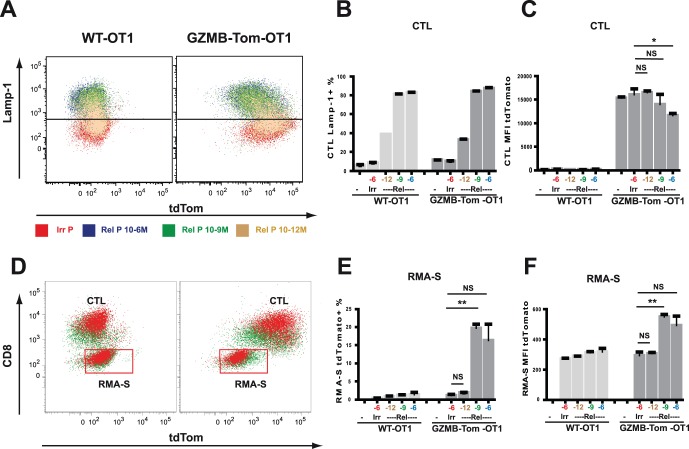
Lamp-1 externalization and GZMB-Tom degranulation during CTL activation. OT1 CTL from B6 (WT) and from GZMB-Tom-KI/KI mice were prepared from LN CD8 T cells by antigenic triggering and expanded with IL-2 as described in Materials and Methods. To measure Lamp-1 externalization, 10^5^ CTL were stimulated for 2h at 37°C with RMA-S target cells pre-loaded with the relevant OVA peptide (Rel P) at different concentrations (10^−6^–10^−12^ M) or Irrelevant peptide (Irr P) at 10^−6^ M. Effector to target ratio was 3/1. a-Lamp-1 Ab was added to the activation medium (see Materials and Methods). FACS analysis was performed on cells gated as CD8 positive (CTL). One experiment with concordant duplicates is shown, and is representative of at least 3 experiments. A: Overlays of the analysis are represented for Lamp-1 versus GZMB-Tom for WT and GZMB-Tom-KI/KI OT1 CTL. **B** and **C**: Quantification of the experiments as % CTL positive for lamp-1 mAb uptake (B) and CTL tdTom Mean Fluorescence Intensity (MFI) (C). D: Overlays of the analysis are represented for all cells including CTL and RMA-S cells and are plotted as CD8 versus GZMB-Tom fluorescence. Quantification of the experiments gated on the RMA-S target cells as % cells positive for tdTomato (E) and tdTom MFI (F). Statistics are shown for values of Lamp-1 externalization for CTL incubated with RMA-S targets pre-loaded with different concentrations of relevant peptide versus irrelevant peptide (C), as well as for acquisition of tdTomato fluorescence as % (E) and as MFI (F) by RMA-S targets pre-loaded with different concentrations of relevant peptide versus irrelevant peptide. P<0.01 (**); P<0.05 (*); P>0.05 (NS) (see Materials and Methods).

When CTL are activated by their cognate target cells, cytolytic granules move towards the CTL/target contact zone, by a process induced by LFA-1/ICAM and TCR/Antigen signaling. This leads to granule migration along microtubules [32], their docking at the cytoplasmic membrane [47], and their fusion with the membrane before degranulation of their contents. As a consequence, Lamp-1 becomes exposed at the external face of the CTL plasma membrane where it is accessible to anti-Lamp-1 Ab. Lamp-1 exposure is thus considered to reveal degranulation events [34]. We first asked whether we could observe GZMB-Tom degranulation concomitant with exposure of Lamp-1 during CTL-target cell interaction (Fig. 5A-C). We next analyzed whether GZMB-Tom could be detected within the cognate target cell (Fig. 5D-F).

OVA peptide SIINFEKL presented by RMA-S, a Tap-1−/− H2^b^ tumor cell line, is a strong stimulus for OT1 CTL. FACS data in Figure 5A show Lamp-1 exposure on WT-OT1 or GZMB-Tom-KI/KI-OT1 CTL (see Materials and Methods) incubated with RMA-S target cells pre-loaded with 10^−6^–10^−12 ^M OVA peptide (relevant peptide) or with 10^−6 ^M pKB1 (irrelevant peptide) at an effector/target ratio of 3/1 for 2h. For analysis of the CTL, gating was on CD8 positive cells. A peptide dose dependent increase in Lamp-1 exposure was observed for both WT-OT1 and GZMB-Tom-KI/KI-OT1 CTL in response to the relevant peptide (quantification shown in Fig. 5B). For GZMB-Tom-KI/KI-OT1 CTL, the increase in Lamp-1 exposure (Fig. 5A, B) is correlated with a decrease in tdTom fluorescence (Fig. 5A, C) as compared to the level observed in the presence of irrelevant or low concentration relevant, with decrease in tdTomato MFI reaching significance only for the highest concentration of relevant peptide (Fig. 5C). No tdTomato fluorescence was observed for the WT-OT1 CTL, as expected.

Data in Figure 5D report tdTom fluorescence in the CTL (CD8 positive) and in the RMA-S target cells (CD8 negative) from the same experiment. An increase in tdTom fluorescence is observed in the RMA-S cells loaded with 10^−9^ M and 10^−6^ M relevant peptide, as compared to those incubated with 10^−6^ M irrelevant peptide, and this increase is observed only for the RMA-S cells incubated with GZMB-Tom-KI/KI-OT1 CTL and not for those incubated with WT-OT1 CTL or with GZMB-Tom-KI/KI-OT1 CTL lacking perforin (Perf-KO-GZMB-Tom-OT1; Fig.S4). Statistical analysis (Fig. 5E-F) indicates that up to 20% RMA-S showed increased tdTomato fluorescence (with MFI tdTomato increasing from 300 to 600) for cells loaded with 10^−9^ M relevant peptide. For cells incubated with 10^−6^ M relevant peptide, we noticed that fewer cells remained in the “live cell” gate (results not shown) and, therefore, we may have lost some of the target cells that had taken up GZMB-Tom.

In conclusion, we observed that the GZMB-Tom protein is specifically released from GZMB-Tom-KI/KI CTL during the degranulation process in response to target cells presenting the relevant antigen, which appear to acquire at least part of the released fluorescence by a process requiring perforin. We next asked whether GZMB-Tom is degranulated as an active enzyme.

### GZMB-Tom is Degranulated as a Functional GZMB Protein

**Figure 6:**
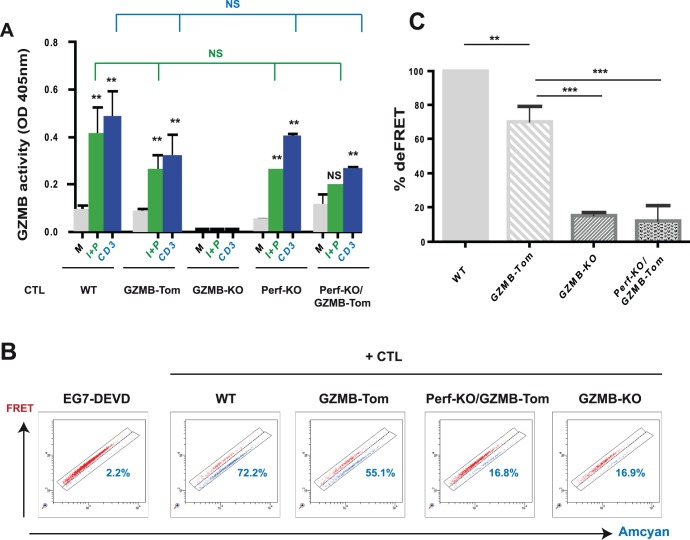
Protease activity of GZMB-Tom degranulated from GZMB-Tom-KI/KI CTL. **A**: OT1 CTL from WT, GZMB-Tom-KI/KI (GZMB-Tom), GZMB-KO, Perf-KO and Perf-KO-GZMB-Tom-KI/KI (Perf-KO/GZMB-Tom) mice were harvested 6 days after stimulation as in Fig. 5. 210^6^ cells in 100 µl medium were incubated in medium (M) or were activated with Ionomycin 2 µg/ml and PMA 50 ng/ml (I+P) or with coated a-CD3 (CD3) for 4h to induce CTL degranulation. Supernatants were assayed for GZMB protease activity after GZMB capture on a-GZMB-coated plates (kit QuickZyme Biosciences) according to the *manufacturer’s protocol*. GZMB activity is reported as optical density at 405 nm for sample minus background. One experiment representative of three with similar results is presented. Statistics are shown for values of GZMB activity measured after activation with I+P or with a-CD3 versus medium for each CTL, as well as for genetically-modified CTL versus WT CTL for either I+P (green lines) or a-CD3 (blue lines) activation. P values as in Fig. 5. **B and C**: GZMB-Tom activity measured in EG7-DEVD target cells during incubation with CTL. OT1 CTL from WT, GZMB-Tom-KI/KI (GZMB-Tom), Perf-KO-GZMB-Tom-KI/KI (Perf-KO/GZMB-Tom) and GZMB-KO mice were prepared from LN CD8 T cells by antigenic stimulation in culture and expansion with IL-2 (see Materials and Methods). Targets cells are EG7-DEVD, EL4 cells expressing OVA as well as the FRET-based fluorescent probe CFP-DEVD-YFP (see Materials and Methods). EG7-DEVD cells were incubated alone or in the presence of the various types of CTL (+ CTL) at 1/1 ratio (10^5^ cells each) for 1h at 37°C. FACS analysis represents the FRET fluorescence of the probe versus CFP fluorescence with discrimination of two separate zones on the diagonal (see Materials and Methods). The upper zone represents the FRET signal of the un-cleaved probe, while the lower one corresponds to FRET disruption after cleavage of the probe. The % of cells in the deFRET zones is indicated on the graphs (B). Statistical analyses from 2 experiments at effector to target ratios from 0.3–3/1 are shown in (C) as % deFRET with deFRET for the WT CTL set at 100%. Results are from more than 3 experiments for WT and GZMB-Tom CTL, and two experiments for GZMB-KO and Perf-KO-GZMB-Tom CTL. Statistics are shown for % deFRET comparing WT, GZMB-KO and Perf-KO/GZMB-Tom CTL with GZMB-Tom CTL. P values as in Fig. 5.

In the following series of experiments we asked whether GZMB-Tom is active as a protease (i) after its degranulation from the CTL and (ii) within the cognate target cells. In order to test whether GZMB-Tom is degranulated as an active enzyme, CTL were reactivated with plastic-coated anti-CD3 mAb or Ionomycin and PMA for 4h and supernatants were assayed for GZMB-dependent protease activity (see Material and Methods). In addition to the WT-OT1 and GZMB-Tom-KI/KI-OT1 CTL, we tested GZMB-KO-OT1 CTL as a negative control and Perf-KO-GZMB-Tom-KI/KI-OT1 CTL to further evaluate the role of perforin with respect to GZMB delivery. Results (Fig. 6A) showed that supernatants from non-activated WT or GZMB-Tom-KI/KI CTL contained low protease activity (0.17 and 0.10 units, respectively), whereas those from Ionomycin/PMA or anti-CD3 mAb stimulated CTL contained, respectively, about 3-fold and 4 to 5-fold increased activity for both WT and GZMB-Tom-KI/KI CTL, while all supernatants from GZMB-KO CTL were negative. The Perf-KO CTL, whether expressing the WT or GZMB-Tom protein, were able to degranulate an active GZMB. Note that we always detected a slightly lower GZMB activity for the GZMB-Tom-KI/KI. This may be due to a lower recognition of the fusion protein GZMB-Tom by the anti-Human GZMB mAb provided with the GZMB detection kit. Similarly, anti-Human GZMB used in FACS analysis (Fig. 3B) appeared to detect the GZMB-Tom protein less well than the WT GZMB.

This experiment showed that CTL from GZMB-Tom-KI/KI mice were able to degranulate an enzymatically active GZMB-Tom fusion protein to the same extent as CTL from WT mice.

### GZMB-Tom Protein is an Active Protease after its Delivery by CTL into Cognate Target Cells

To evaluate the activity of GZMB-Tom within target cells we used the EG7-DEVD target cells described by Breart and colleagues [41]. They express a FRET-based CFP-DEVD-YFP probe such that cleavage of the DEVD sequence directly by GZMB or indirectly via active caspase 3 results in loss of the FRET signal (deFRET) that can be detected by FACS analysis (see Materials and Methods and legend to Fig. 6B). FACS analyses show EG7-DEVD target cells incubated for 1h in medium alone or in the presence of various OT1-based CTL (Fig. 6B). No deFRET was observed in targets incubated alone (2.2%, not shown), whereas significant deFRET was induced by both WT-OT1 CTL (72.2%) and GZMB-Tom-KI/KI-OT1 CTL (55.1%). Importantly, GZMB-KO-OT1 CTL induced only a small deFRET (16.8%), suggesting that most of the activity detected at 1h is dependent on the incoming GZMB-Tom. Further, the level of deFRET induced by Perf-KO CTL (not shown) or Perf-KO-GZMB-Tom-KI/KI CTL (16.8%) was similar to that induced by the GZMB-KO-OT1 CTL (16.9%; Fig. 6B-C). These results suggested that the main deFRET activity was due to the activity of GZMB or GZMB-Tom, in a perforin-dependent manner.

### Direct Visualization of Distinct Steps of Cytolytic Function Activation using CTL from GZMB-Tom-KI/KI Mice

**Figure 7:**
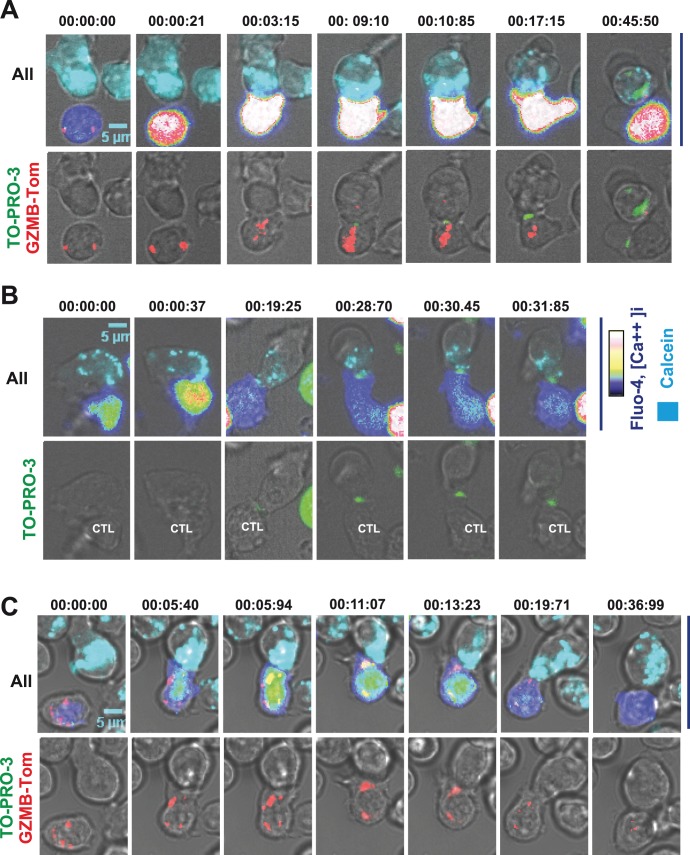
Visualization of the kinetics of CTL activation, granule relocalization and target cell death. OT1 CTL from GZMB-Tom-KI/KI (A), WT (B) or Perf-KO-GZMB-Tom-KI/KI (C) prepared as in Fig. 7 were labeled with 2.5 µM Fluo-4. For video microscopy analysis, RMA-S target cells pre-incubated with 1 µM OVA peptide and loaded with 1 µM Calcein AM were deposited onto poly-lysine activated Labtek wells, before the addition of the Fluo-4 labeled CTL as described in Materials and Methods. TO-PRO-3 was present in the medium (see Materials and Methods). Fluorescence signals from GZMB-Tom (A, C), Fluo-4 (reported as rainbow RGB false color) and calcein (cyan) as well as brightfield (A, B, C) were recorded every 12.5 sec for around 90 min. Image J or ZEN software was used for image analysis. Images reporting all 4 signals (upper A; upper C), GZMB-Tom (red), TO-PRO-3 (green) and brightfield (lower A; lower C) or 3 signals (upper B) and TO-PRO-3 (green) and brightfield (lower B) are shown for selected times. Videos are provided as Supporting Information. Statistical analysis of the different events observed in those videos are reported in Fig.S5 and S6.

To visualize the distribution of GZMB containing granules at different steps of CTL/target cell interactions, we monitored the encounter of GZMB-Tom-KI/KI-OT1 CTL with their cognate target cells (OVA peptide pulsed RMA-S cells - see Materials and Methods). CTL were loaded with Fluo-4 to detect Ca^++^ signaling in the CTL while the target cells were pre-loaded with Calcein Violet-AM (see Materials and Methods) to detect dye leakage upon damage of the target plasma membrane. TO-PRO-3 was present in the medium throughout the experiment, allowing for detection of its binding to DNA of apoptotic target cells after its entry into target cells suffering plasma membrane permeabilization. Results are shown (Fig. 7A and Video S1) for a GZMB-Tom-KI/KI-OT1 CTL and, as a control, for a CTL from a similar experiment performed with WT-OT1 CTL (Fig. 7B) and for a Perf-KO-GZMB-Tom-KI/KI-OT1 CTL (Fig. 7C and Video S2). The time at which different events occur for each observed conjugate has been compared for the different types of CTL (Fig.S5).

In figure 7A, images show (time 0) one CTL (blue-green Fluo-4 signal; un-polarized red granules) in contact with one target (calcein labeled). Twenty one sec later, the first Ca++ signal ([Ca^++^]i increase to red) is observed in the CTL. In the next image (3 min 15 sec) a tight association between CTL and target cell is observed with the CTL showing a further increase in [Ca^++^]i and polarization of its red granules towards the center of the CTL/target contact zone, as previously shown [39], [48]. Surprisingly, in the next image (9 min 10 sec) a ring of TO-PRO-3 (in green Fig. 7A) appeared at the CTL/target contact zone, in front of the relocalized granules. Note that a similar “TO-PRO-3 ring” appeared in the experiment using the control WT-OT1 CTL (starting at 19 min 25 sec, Fig. 7B). At the same time, the calcein labeling, which is mostly localized in vesicles in the RMA-S target cells, started to leak out of the target cell cytoplasm (at 9 min 10 sec in Fig. 7A, and 19 min 25 sec in Fig. 7B). The TO-PRO-3 ring increased in size and fluorescence intensity until the target’s apoptotic nucleus next became labeled with TO-PRO-3 (from 37 min 45 sec, shown at 45 min 50 sec in Fig. 7A, and 31 min 85 sec in Fig. 7B). At this time, the GZMB-Tom-KI/KI-OT1 CTL appeared to have lost most of its GZMB-Tom red granules (Fig. 7A).

Red GZMB-Tom “spots” were occasionally (in <10% of the conjugates – see Fig.S5) observed in the target cell (Fig. 7A, 9 min 10 sec and 45 min 50 sec). Their significance is not clear, however. In particular, no punctate GZMB-Tom fluorescence was observed in the target cell at the contact zone with the CTL. If GZMB-Tom were delivered “free” in the target cell cytoplasm, it would not be possible to visualize it at the video confocal microscope. If, however, GZMB is delivered in endosomes in the target cell, as suggested by previous studies [9], [36], [49], we would have expected to detect a punctate tdTom fluorescence signal in the target cell. Whether the few red fluorescent spots observed in these experiments correspond to such endosomes requires further analysis.

The kinetics and sequence of events (increase in CTL [Ca^++^]i, loss of calcein from the target cell, TO-PRO-3 ring and apoptosis) were similar for GZMB-Tom-KI/KI-OT1 and WT-OT1 CTL, (Fig. 7A-B, Fig.S5) indicating that expression of the GZMB-Tom fusion protein does not perturb any of these events. Perf-KO-GZMB-Tom-KI/KI CTL were efficiently activated by the cognate target cells as observed by the increase in [Ca++]i and the granule polarization to the CTL/target zone. As expected, they failed to induce calcein leakage from the target cell. We also did not observe the “TO-PRO-3 ring” and the nuclear TO-PRO-3 uptake (Fig. 7C).

In conclusion, these observations may shed light on some of the events pertaining to the mechanisms of transfer of the contents of cytolytic granules from the CTL to target cells. We could clearly observe the occurrence of a TO-PRO-3 ring at the CTL/target cell contact zone where the CTL granules are relocated and where calcein appears to leak out of the target cell cytoplasm. These results highly suggest the formation of pores in the target plasma membrane at the immune synapse (analysis – Fig.S6). This phenomenon depended on the presence of perforin upon CTL/target cell interaction and was not observed upon CTL activation by anti-CD3-coated beads (results not shown). At this stage it is not clear which cellular component is associated with the fluorescence signal of TO-PRO-3, which is known to acquire its intense fluorescence upon binding to nucleic acids, and DNA in particular. Further experiments are needed to fully understand these observations.

### GZMB-Tom-KI CD8 T Cells as a Probe for in vivo Acquisition of Cytolytic Function

**Figure 8:**
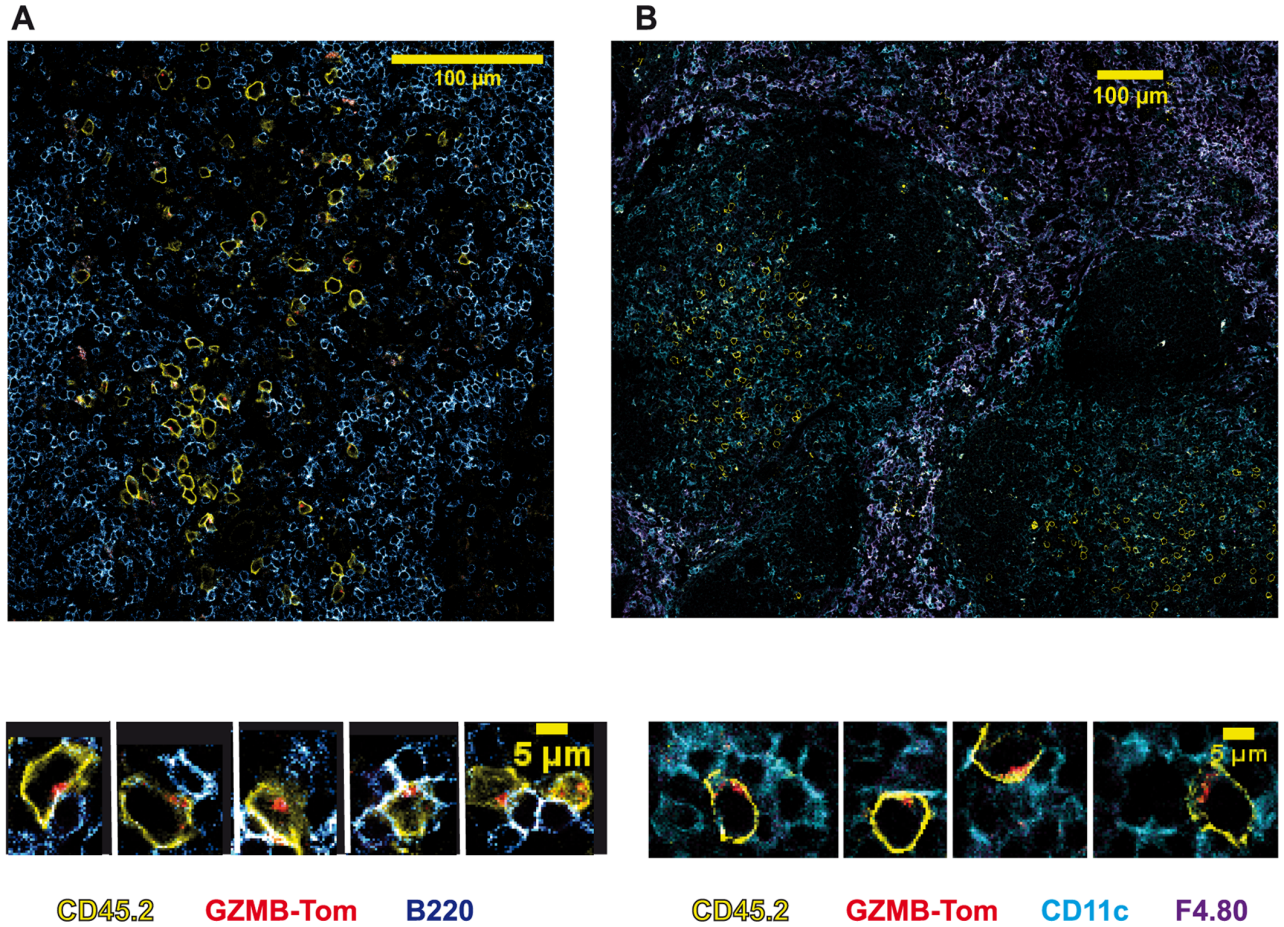
In vivo acquisition of GZMB-Tom expression by GZMB-Tom-KI/KI-KI-OT1 CD8 T cells in response to infection. Naive CD45.1 B6 mice were used as recipients of 3.10^6^ naive CD8 T cells from GZMB-Tom-KI/KI-OT1 CD45.2 mice. Mice were i.v. injected 11h later with 10.000 U Listeria-OVA. Immunohistology (see Materials and Methods) on a spleen section from mice collected 48h after infection shows staining of transferred GZMB-Tom-KI CD8 T cells (CD45.2, yellow) with GZMB-Tom (red) and either staining for B cells (B220, blue) (A) or for CD11c (cyan) and F4.80 (purple) (B). Magnifications highlight the location of the GZMB-Tom containing granules.

We next evaluated the in vivo acquisition of GZMB expression by CD8 T cells during an immune response to an infectious agent. For this, recipient mice (C57BL/6, CD45.1) were injected i.v. with 3.10^6^ naïve GZMB-Tom-KI-OT1 CD8, CD45.2 T cells. The mice were then immunized i.v. with Listeria-OVA (List-OVA, 10.000U) 11h later (Immune, see also Materials and Methods). Immunohistology was performed on sections of the spleens 48h after infection. Images from confocal microscopy (Fig. 8) show the transferred CD45.2 CD8 T cells (yellow) in the splenic white pulp surrounded by the B cell zone (B220 staining) (Fig. 8A-left side image), as well as CD11c positive DC (cyan) in the same white pulp zone (Fig. 8B-right side image) with F4.80 positive macrophages (purple) surrounding the white pulp. By cropping on GZMB-Tom positive cells, we can see that at least some of them appear to be in conjugates with CD11c positive cells and to present polarized GZMB-Tom containing granules. It was reported that many splenic DC present OVA to T cells in the white pulp zone at this time after immunization with List-OVA [45]. It is not clear, however, whether at this early time point the granule polarization reveals active cytolytic events or whether the CD8 T cells are in the course of differentiation.

In conclusion, the GZMB-Tom-KI (KI or KI/KI) mouse is an innovative tool for in vitro and in vivo analysis of GZMB acquisition during an immune response. The in vivo visualization of GZMB-Tom in redistributed granules suggests that this is a powerful model to further analyze cytolytic function expression in normal and pathological situations.

## Discussion

We have previously shown that a construct containing *Gzmb* cDNA deleted of the stop codon, with an added sequence encoding a 12 amino acid linker followed by the *tdTomato* gene led to the expression of a red fluorescent fusion protein in transduced CTL. This GZMB-Tom protein had a MW around 80kDa and was localized in Lamp-1 positive granules of these cells [39]. Obvious limitations of approaches based on over-expression of a fusion protein and low efficiency in T cell transduction led us to generate a genetically modified mouse that expresses the same GZMB-Tom fusion protein in place of GZMB, maintaining the gene expression control elements of the endogenous *gmzb* gene. This GZMB-Tom-KI/KI mouse presents the additional advantage of allowing in vivo studies of the acquisition of GZMB expression in various cells participating in the immune response.

In the present report we described the construction of the GZMB-Tom-KI/KI mouse and show that GZMB-Tom faithfully reports the expression of endogenous GZMB in T cells in unchallenged mice, as well as upon transfer in Listeria monocytogenes-infected mice. The latter experiment used OVA-specific GZMB-Tom-KI-OT1 CD8 T cells, which acquired GZMB-Tom expression as soon as 48h after infection with OVA-Listeria monocytogenes. Interestingly, confocal microscopy of spleen sections revealed GZMB-Tom positive CD8 T cells interacting with CD11c positive DC, with their GZMB-Tom-containing granules redistributed to the T/DC contact zone. One previous report elegantly showed perforin expression by CTL interacting with LCMV-infected cells in brains of infected mice [50]. Such studies required staining with anti-perforin mAb on fixed and permeabilized tissue samples. The strong fluorescence signal observed for the GZMB-Tom-KI/KI containing granules of CD8 T cell early after infection in mice indicates that it will be possible to monitor changes in granule distribution in effector and memory CD8 T cells. Comparison of conditions leading to productive secondary responses versus situations of chronic stimulations leading to some form of “exhaustion” will be most informative.

Since GZMB is an important component of the cytolytic machinery of CD8 T cells and NK cells, we first concentrated our efforts on showing that the GZMB-Tom fusion protein retained protease activity upon its degranulation from CTL in supernatants and also within the CTL cognate target cells. In particular since the fusion protein is much larger (80 kDa) than the native GZMB (29 kDa), it was important to show that the distribution, capacity to exocytose and to enter the target cells were retained. All of the assays performed with CD8 T cells from homozygous GZMB-Tom-KI/KI mice in which the fusion protein is the only source of GZMB indicate that the fusion protein behaves as native GZMB, retaining its protease activity when delivered within the target cells.

The finding that the GZMB-Tom faithfully presented all properties of native GZMB allowed us to evaluate for the first time (i) the movements of undisturbed cytolytic granules by time-lapse video microscopy and (ii) the delivery of GZMB-Tom at the immune synapse of CTL interacting with their cognate target cell. The first point was previously approached using over-expression of a granule component [37], [39] or use of a lysotracker [51], whereas the second point was generally addressed by addition of GZMs to target cells in the presence of sub-lethal doses of perforin [37].

Two main paradigms remain concerning the mechanism by which perforin allows GZMs to access the target cell’s cytoplasm. (i) In the plasma membrane pore forming model, GZMs access from the synaptic cleft through pores formed in the target cell plasma membrane by perforin [52], as initially suggested [53], [54]. (ii) An alternative “internalization” model proposes that GZMs and perforin are internalized via endocytosis at the target cell plasma membrane into large endosomes (gigantosomes) as a result of a membrane repair mechanism in response to damage induced by perforin to the plasma membrane. After internalization perforin would form larger pores in these endosomes’ membranes thereby delivering the GZMs to the target cell’s cytoplasm [37]. Most of the latter studies involved addition of GZMs to target cells in the presence of sub-lethal doses of perforin or NK cells overexpressing GZMB [37].

In the present study, visualization of endogenously fluorescent GZMB could be combined with fluorescent dyes tracing activation in the CTL and perturbations of the target cell.

Although formation of a stable immune synapse between CTL and target cell may not be required for CTL cytolytic activity [55], high avidity CTL/target cell interactions such as those involving the CD8+ OT1 CTL/OVA peptide-loaded target cell system used in our study have been shown to lead to granule polarization to the immune synapse [56]. Accordingly, in our live video experiments, we observed the movement of the red GZMB-Tom granules towards the CTL/target cell tight interaction zone corresponding to the immune synapse. Intriguingly, when the TO-PRO-3 dye was present in the medium, a fluorescent TO-PRO-3 signal appeared at this junction between the CTL and the target cell several minutes before a fluorescent signal was visible in the target cell nucleus. A similar biphasic TO-PRO-3 signal, first proximal to the CTL/target cell contact zone, later nuclear, was also observed for WT CTL. In CTL expressing GZMB-Tom, but not perforin, red granule polarization towards the CTL/target cell synapse was readily observed, but it was not followed by a fluorescent TO-PRO-3 signal either at the immune synapse, or in the target cell nucleus. When GZMB-Tom KI/KI CTL were induced to degranulate by anti-CD3 coated beads, no TO-PRO-3 ring appeared at the CTL/bead interface (results not shown). Altogether, these observations suggest that a perforin-dependent event in the cleft between CTL and target cell allows the entry of TO-PRO-3 (MW 671) generating the target cell membrane proximal fluorescent signal. As the fluorescence quantum yield of TO-PRO-3 is greatly enhanced upon binding of the dye to DNA, and to a lesser extent to RNA, it is not clear whether the membrane proximal fluorescence signal corresponds to a high concentration of free dye, or to dye bound to some fluorescence enhancing compound. Similarly, evidence for perforin-dependent permeabilization of fibrosarcoma target cells at the synaptic cleft was recently proposed on the basis of the observation of local diffusion of extracellular propidium iodide (MW 668) into target cells [57]. The question as to whether in addition to thymoma lines (our study) and fibrosarcomas cells [57], similar early plasma membrane permeabilization events occur for tumor cells of different origins, and particularly those that appear more resistant to CTL action, such as melanomas [58], will be further investigated. Different mechanisms may include distinct responses (i) to the initial perforin-dependent permeabilization, as target cells may be differentially equipped to repair plasma membrane pores [9], [37]; (ii) to cell death effector molecules [8].

The fluorescent GZMB-Tom also allows the quantification of the amount of GZMB that is exocytosed from the CTL and that enters the target cell. Upon degranulation almost all the CTL expose Lamp-1 and lose around 35% of GZMB-Tom (this is similar to the decrease in GZMB in WT CTL, not shown). In comparison, around 3% of exocytosed GZMB-Tom could be detected in the RMA-S target cells. This amount may be underestimated as FACS gating was on live target cells. The fact that small amounts of GZMs would be delivered in individual target cells is consistent with the serial killing capacity of CTL [59] and the high efficiency of the cytolytic function [60]. In addition, the synapse cleft has to be leaky, as shown by the access of a-Lamp-1 Ab to externalized Lamp-1 [35] and some of the GZMB probably leaks out of the cleft. Our study showed that the small amount of GZMB-Tom delivered in the target cells was sufficient to induce GZMB-dependent cleavage of the deFRET probe.

The pore size of polymerized perforin was recently observed and estimated [6], [61] to have a 16 nm diameter, compatible with entry of 29kDa GZMB, 50–60kDa dimer GZMA and 80kDa GZMB-Tom. Using T lymphoma target cells (RMA-S/EL4) and CTL with a high affinity TCR for its cognate peptide-MHC, spots containing fluorescent GZMB-Tom before target cell death were detected in fewer than 10% of the observed CTL/target cell conjugates in the video experiments (Fig. 7, Fig.S5). This means that in most cases internalized GZMB-Tom in the target cell, that could be detected by FACS analysis, was not localized in vesicles as proposed by other authors [9] [37]. In addition, no GZMB-Tom vesicles could be detected when CTL where from Perf-KO-GZMB-Tom-OT1 mice. If GZMB internalization was independent of perforin, as initially proposed [49], an accumulation of GZMB-Tom in vesicles in the target cells would have been expected in the absence of perforin, as internalized GZMB-Tom would fail to be released from the vesicles.

Our data are thus compatible with a perforin-dependent permeabilization event in the target cell plasma membrane allowing entry of the dye TO-PRO-3 at the synaptic cleft as well as access of GZMB-Tom to the target cell’s cytoplasm.

As different target cells may respond differentially to an initial membrane damage, similarly CTL effector cells may vary in the number and content of their granules depending on their differentiation status, and they may differ in the strength of TCR stimulation as well. Both these parameters may control the actual concentration of granule contents in the synaptic cleft, possibly leading to either (i) strong plasma membrane damage, or to (ii) weak damage more amenable to repair. Visualization of GZMB-Tom transfer using different CTL/target cell characteristics may help resolving these issues.

In summary, we describe a new tool that allows monitoring of in vivo expression and activation steps of granule containing CTL and NK cells. It will also permit evaluation of GZMB expression in other cell populations such as mast cells, dendritic cells and regulatory T cells, all of which have been reported to express GZMB under some normal or pathological situations.

## Supporting Information

Figure S1 **Construction of the GZMB-Tom-KI mouse. A**: Modification of the *gzmb* locus containing BAC clone (RP23-38D2) in DH10β by homologous recombination and digestion/ligation. Two small fragments of BAC DNA (black bars; A box and B-box) were amplified by PCR and incorporated into a target vector (pBS-KS) to act as homologous sequences for recombination. Fse I, Not 1, Eco RV and Sal I sites were included by homologous recombination to allow the incorporation of another DNA fragment (extracted from a previous construct). The inserted construct contains the last 5′ exon of the *gzmb* gene, deleted of its stop codon and added to a sequence encoding a 12 amino acid linker followed by the *tdTomato* cDNA sequence. A self-deleter neo resistance cassette and a counter selection TK sequence were inserted by digestion/ligation as a requirement for the final homologous recombination in B6 ES cells. **B**: Schematic maps of the recombinant locus in the homologous recombination vector (***Gzmb-tdTomato Neo***) and the final locus (***Gzmb-tdTomato*** **Δ** ***Neo***) in the *Gzmb-tdtomato* Knock-In (KI) mouse after deletion of the NEO cassette. **C**: Final verifications of recombinant ES clone were performed. 5′ and Neo screens were performed by southern blot. The 3′ screen was performed by long range PCR (see Materials and Methods).
(EPS)

Figure S2 **Immunoblot characterizing the GZMB-Tom fusion protein in GZMB-Tom-KI CTL.** NP40 lysates of 5.10^6^ CTL from WT, GZMB-Tom-KI/KI and GZMB-Tom-KI mice were immunoprecipitated with the anti-RFP Ab from the Rockland Western Blot Kit. A 7–17% acrylamide gradient in reduced conditions was performed, before blotting onto Immobilon P in CAPS Buffer [39]. The immunoblot was revealed with the same a-RFP Ab and a-Rabbit-Ig-HRP from Rockland Kit. A LAS1000 was used to reveal and measure chemoluminescence.
(EPS)

Figure S3 **Statistics for evaluation of colocalization of tdTom fluorescence with GZMB, GZMA and Lamp-1.** Colocalization of fluorescence markers shown in Fig. 4 was analyzed using Image J software. Rr Pearson’s coefficients are shown for a number of isolated resting GZMB-Tom-KI CTL (A-C) and for CTL/target cell conjugates (D) as in Fig. 4. Colors: red (R), green (G), blue (B) as in Fig. 4.
(EPS)

Figure S4 **Lamp-1 externalization and GZMB-Tom degranulation during activation of Perf-KO- GZMB-Tom-KI/KI CTL.** OT1 CTL from Perf-KO-GZMB-Tom-KI/KI mice were prepared and incubated with irrelevant peptide or relevant OVA peptide loaded RMA-S target cells and stained with a-Lamp-1 and a-CD8 mAb as described in Fig. 5. Overlays of the FACS analysis of Lamp-1 versus tdTom are represented for CD8 positive CTL (A) and of CD8 versus tdTom for all cells including CTL and RMA-S cells (B). Values for tdTom MFI and % Lamp-1 positive cells are indicated (A) when gating on the CD8-positive CTL. Values for tdTom MFI when gating of the RMA-S target cells are also shown (B).
(EPS)

Figure S5 **Summary of the different steps analyzed in CTL activation using video microscopy (** **Fig. 7** **).** Results from at least 3 experiments for OT1 CTL from WT and GZMB-Tom-KI/KI and 2 from Perf-KO-GZMB-Tom-KI/KI mice are represented. The timing for all events was adjusted on the Ca++ signal, which is set as time 0. For the WT CTL, as they do not express GZMB-Tom, there is no report of granule polarization (Gran Pol). For most conjugates from the Perf-KO-GZMB-Tom-KI/KI CTL there was no TO-PRO-3 “ring” and no nuclear TO-PRO-3, neither was there target cell death nor calcein release (Calcein rel). GZMB-Tom red spots in target cells (Target Gtom+) were occasionally detected (5/58 events) only with GZMB-Tom-KI/KI OT1 CTL.
(EPS)

Figure S6 **Analysis of GZMB-Tom and TO-PRO-3 distribution in CTL/target cell conjugates.** Images of conjugates of OT1 CTL from GZMB-Tom-KI/KI (A, B) and from WT (C) mice with OVA-peptide-loaded RMA-S target cells, labeled and analyzed as in Fig. 7, are shown at times when TO-PRO-3 fluorescence becomes visible at the synaptic cleft (left images) and at a later time (right images). Fluorescence histograms were measured along the white arrows using the Zen software. The blue and the red profiles depict, respectively, the Fluo-4 and the GZMB-Tom staining in the CTL. Green and cyan profiles depict, respectively, the TO-PRO-3 and calcein staining. The left-side histograms show the positioning of a TO-PRO-3 signal in front of the GZMB-Tom signal towards the target cells before any signal is detected in the target cell nucleus. At later time points (right-side histograms), a bimodal distribution of TO-PRO-3 is generally observed, one proximal to the target plasma membrane, the other nuclear. In (C) the analysis shows the distribution of Fluo-4, TO-PRO-3 and calcein for a WT CTL/target cell conjugate with TO-PRO-3 fluorescence at the CTL/target contact zone (left) and diffused in the target cell (right).
(EPS)

Video S1 **Kinetics of activation of OT1 CTL from GZMB-Tom-KI/KI mice.** Conditions are described in Legend to Figure S5.
(AVI)

Video S2 **Kinetics of activation of OT1 CTL from Perf-KO-GZMB-Tom-KI/KI mice.** Conditions are described in Legend to Figure S5.
(AVI)
